# *KRAS*-Mutant Non-Small-Cell Lung Cancer: From Past Efforts to Future Challenges

**DOI:** 10.3390/ijms23169391

**Published:** 2022-08-20

**Authors:** Serena Ceddia, Lorenza Landi, Federico Cappuzzo

**Affiliations:** 1 Division of Medical Oncology 2, IRCCS Regina Elena National Cancer Institute, Via Elio Chianesi 53, 00144 Rome, Italy; 2 Clinical Trials Center: Phase 1 and Precision Medicine, IRCCS Regina Elena National Cancer Institute, Via Elio Chianesi 53, 00144 Rome, Italy

**Keywords:** non-small-cell lung cancer, *KRAS* mutations, *KRAS*G12C inhibitors, molecular biology, targeted therapy

## Abstract

*KRAS* is the most frequently mutated oncogene identified in human cancers. Despite the numerous efforts to develop effective specific inhibitors against *KRAS*, this molecule has remained “undruggable” for decades. The development of direct KRAS inhibitors, such as sotorasib, the first FDA-approved drug targeting *KRAS* G12C, or adagrasib, was made possible with the discovery of a small pocket in the binding switch II region of *KRAS* G12C. However, a new challenge is represented by the necessity to overcome resistance mechanisms to KRAS inhibitors. Another area to be explored is the potential role of co-mutations in the selection of the treatment strategy, particularly in the setting of immune checkpoint inhibitors. The aim of this review was to analyze the state-of-the-art of *KRAS* mutations in non-small-cell lung cancer by describing the biological structure of *KRAS* and exploring the clinical relevance of *KRAS* as a prognostic and predictive biomarker. We reviewed the different treatment approaches, focusing on the novel therapeutic strategies for the treatment of *KRAS*-mutant lung cancers.

## 1. Introduction

In non-small-cell lung cancers (NSCLCs), identifying molecular mechanisms underlying tumor growth and progression is crucial to defining the best therapeutic approach for each patient. In recent years, great strides have been made to improve the individualized therapeutic approach for each patient. Among the molecular alterations predicting response to targeted treatment in lung cancer, epidermal growth factor receptor (EGFR) inhibition was the first to succeed [1]. Studies have shown that in patients with molecular rearrangements and specific mutations (e.g., *EGFR*, *ALK* and *ROS1*), targeted therapy with tyrosine kinase inhibitors (TKIs) leads to a significant improvement in survival and quality of life [2]. Some new molecules have shown efficacy in inhibiting rare oncogenic drivers, such as *RET*, *BRAF*, *MET*, *NTRK* and *HER2* [3]. *KRAS* is the most common alteration identified in solid tumors and especially in pancreatic (88%), colorectal (45–50%) and lung cancers (31–35%) [4]. G12D, G12V, G12C, G13D and Q61R are five different mutations that account for 70% of all *RAS*-mutant patients. *KRAS* G12C mutations are frequently found in lung cancer due to G:C>T:A transversions associated with carcinogen-DNA adduct generated by the mutagens in tobacco smoke [5].

Given the high frequency of *KRAS* mutations in different types of aggressive tumors, numerous efforts have been made to block the function of this oncoprotein.

*KRAS* mutations may be associated with poor overall prognosis and response in advanced/metastatic NSCLC [6]. Traditionally, the standard treatment for patients with *KRAS* mutations in NSCLC is chemotherapy, and the average overall survival (OS) rate is less than 2 years [7]. Several studies showed that *KRAS* mutations in patients with NSCLC have a negative impact on OS, progression-free survival (PFS) and disease control rate (DCR) [8,9]; moreover, patients develop liver and brain metastases more frequently in the presence of this mutation and experience a more aggressive form of the disease [8].

Despite numerous attempts over the last three decades aimed at identifying inhibitors of *RAS*, many questions remain unanswered. Recently, small-molecule inhibitors demonstrated great efficacy in the pharmacological inhibition of the *KRAS* p.G12C mutation, as monotherapies and in combination with other treatments. Given these assumptions, the purpose of this review was to explore the biological basis on which the efforts made to investigate indirect inhibition mechanisms and direct targeting strategies of mutated forms of *KRAS* are based.

## 2. Molecular Biological Functions of *KRAS*

The human *KRAS* gene, located on chromosome 12.p12.1, is the most common oncogenic driver, with G12C representing the most frequent mutation in NSCLC, followed by G12V, G12D, and G12A. *KRAS* is present in a mutated form in 80% of pancreatic cancers, 40% of colorectal cancers, and about 30% of lung adenocarcinomas [10]. In NSCLC, *KRAS* mutations are present in approximately 30% of lung adenocarcinomas and 5% of squamous cell carcinomas; these alterations are more common in western (26%) than in Asian (11%) populations and in smokers (30%) than non-smokers (10%) [11,12,13]. The smoking habit has also been related to the type of *KRAS* mutation. Transversion mutations, such as the guanine to thymine mutation, are more common in current or former-smoker patients, while never-smoker patients more frequently have transition mutations [11]. Proto-oncogene *KRAS* encode a GTPase protein involved in extracellular to intracellular signal transduction through activation and inactivation determined by the binding to GTP [14]. The transduction pathway of RAS proteins is involved in intracellular signaling and tumor cell growth [15]. KRAS molecule interactions involved in its signaling include EGFR, MEK, MAPK, Raf, PI3K and AKT. The active form of the protein is represented by the GTP-binding form, while the protein is demonstrated to be inactive in its GDP-binding form. The gamma-phosphate of the GTP analog determines conformational changes in two switch regions of the RAS protein. Downstream effectors interact with the switch I and II regions, held by the gamma-phosphate of GTP. The inactive state, incapable of binding effector molecules, is derived from the hydrolysis of the gamma-phosphate, which determines a structural change in the switch regions.

**Figure 1:**
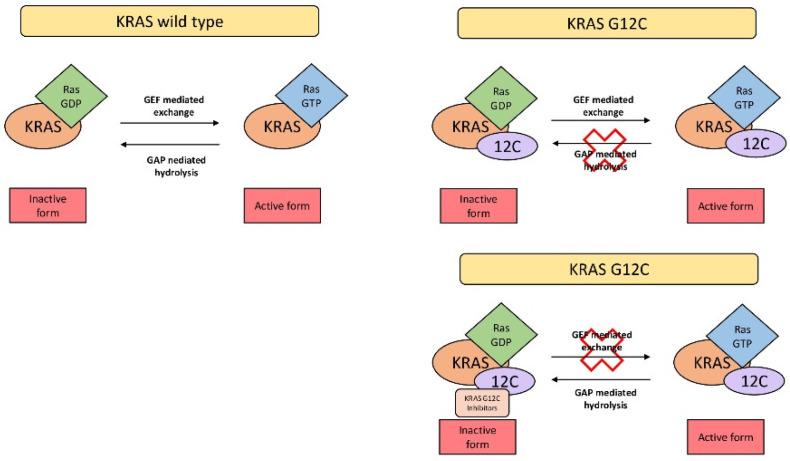
GEFs are required to convert the GDP-bound inactive RAS to the GTP-bound active form. The RAS intrinsic hydrolytic activity allows the conversion back to the GDP inactive form. Missense mutations (such as cysteine 12 mutations) lock *KRAS* in the active state and disable *KRAS’s* function to hydrolyze GTP. KRAS G12C inhibitors can covalently bind to the switch II pocket of KRAS G12C, converting KRAS into an inactive state.

Guanine nucleotide exchange factors (GEFs) are required to convert the GDP-bound inactive RAS to the GTP-bound active form. The RAS intrinsic hydrolytic activity allows for the conversion back to the GDP inactive form (Figure 1).

Both molecules of GTP and GDP have a high binding affinity for RAS. The intrinsic GTPase activity of RAS and the dissociation rates of GDP are low, so high levels of GEFs are required for conversion into the active form of RAS. Therefore, missense mutations lock KRAS in the active state and disable the KRAS’s function to hydrolyze GTP, leading to the constitutive activation of its effector proteins and increased downstream effects such as cell proliferation and survival. The mutation of *KRAS* in codon 12, normally occupied by a glycine residue, leads to a steric block that hinders the binding of GTPase-activating proteins (GAPs) to KRAS, reducing GTP hydrolysis and maintaining high levels of GTP-bound active form [16].

## 3. *RAS*-Mutant Biological Heterogeneity

Intra-tumor heterogeneity and different treatment responses are a current challenge in developing targeted therapies in patients with lung cancer and *KRAS* mutation. Co-occurring molecular alterations in lung cancer have been revealed as a major concept on which the molecular diversity of tumor biology is based [17]. Concomitant mutations, including the *TP53* tumor suppressor gene, serine/threonine kinase 11 (*STK11*) and kelch-like ECH-associated protein 1 (*KEAP1*) have been reported in about half of *KRAS*-mutant NSCLC [18]. The downstream signaling derived from different co-mutation partners leads to variances in genetic events, suggesting a difference in treatment response. Skoulidis et al. [18] explored data from an early stage and chemorefractory disease and examined the various heterogeneity of *KRAS*-mutant NSCLC. The following three *KRAS* subsets were defined based on the presence of co-mutations: *STK11*/*LKB1* (‘KL’), *TP53* (‘KP’) and *CDKN2A*/*B* inactivation (‘KC’). Another critical co-mutation described is *KEAP1*/*NFIE2L2*, which was included in the KL subgroup. Tumors with *TP53* alterations demonstrated an improved overall response rate ([ORR] 7.4% KL vs. 35.7% KP vs. 28.6% *KRAS* mutations alone) [19]. Patients with co-mutations in *KEAP1*/*NFE2L2* have a significantly shorter survival. Co-mutations of *KRAS* with *STK11*/*LKB1* were associated with a lower response to immune checkpoint blockade, poor immune micro-environment, and low PD-L1 expression proved to be immune-inert tumors [19,20]. Instead, *KRAS–TP53* co-mutant tumors expressed high levels of cytotoxic CD-8 Th1 and tumor-infiltrating lymphocytes [18]. These data highlight the heterogeneity of *KRAS*-mutant NSCLC, proposing the predictive value of concomitant genetic events.

## 4. Why Has Targeting *KRAS* Been Difficult?

One of the most challenging and, at the same time, attractive therapeutic targets in cancer is represented by mutant *KRAS*. RAS proteins lack a deep pocket for small molecules to bind with high affinity; high intracellular GTP concentrations and its strong affinity to KRAS brought poor results in the various efforts to find small drugs that bind tightly to RAS proteins. Furthermore, the indiscriminate inhibition and the high blood levels of proteins needed to block KRAS function could lead to great potential toxicity [21,22,23].

Therefore, alternative approaches have been adopted, focusing on the downstream inhibition of KRAS activity. These indirect strategies demonstrated low efficacy explainable by extensive post-transcriptional modifications and the activation of different KRAS signaling pathways, leading to the need to target the RAS protein with different simultaneous strategies [21].

## 5. How to Target *KRAS*-Mutated Lung Cancer

### 5.1. Indirect KRAS Inhibition

#### 5.1.1. Targeting KRAS Membrane Associations

The oncogenic transforming activity of RAS requires farnesylation; interfering with the farnesylation of Ras proteins prevents membrane localization and significantly reduces transformation activity. Several farnesyltransferase inhibitors (FTIs) have been tested to target the post-translational modification of KRAS and disrupt the farnesylation of oncogenic RAS (Table 1).

FTIs showed in vitro activity in *KRAS*-mutant lung cancer in mice [24,25], but its clinical activity has not been confirmed in NSCLC clinical trials [26,27,28].

The unsuccessful strategy to target *KRAS* for cancer therapy through FTIs might be explained by the existence of different *RAS*-mutant isoforms [29]. Preclinical studies showed FTIs failed to inhibit *KRAS* and *NRAS* function due to alternative membrane-binding mechanisms; therefore, only *HRAS*-mutant cancer cells were sensitive to FTIs.

Salirasib inhibits all isoforms of *Ras* in contrast to FTIs, and its efficacy as a single agent was tested in a phase II trial [30]. Salirasib did not show significant antitumor activity in this study, and no partial responses were observed.

Recently, several clinical trials investigated the efficacy of vaccination. mRNA-5671 was examined as monotherapy, and in combination with pembrolizumab in an ongoing phase I trial (ClinicalTrials.gov Identifier: NCT03948763).

**Table 1:** Indirect KRAS inhibition—targeting *KRAS* membrane associations and cancer vaccine.

Target	Therapeutic Drug	Trial	Patients (n)	Overall Response Rate	Ref.
Farnesyltransferase inhibition	Lonafarnib + paclitaxel	Phase II	33	10%	[26]
	Tipifarnib	Phase II	44	0%	[27]
RAS farnesyl cysteine mimetic drug	Salirasib	Phase II	33	0%	[30]
Cancer vaccine	mRNA-5671 + pembrolizumab	Phase I	–	–	NCT03948763

#### 5.1.2. Targeting KRAS-Regulated Pathways

*KRAS* signaling involves various downstream pathways which are potentially targetable by the indirect inhibition of *KRAS*. The dysregulation of the signal induced by *KRAS* mutations includes MET overexpression, the PI3k/AKT/mTOR and the RAF/MEK/ERK pathways [31]. Table 2 summarises the clinical trials examining strategies to prevent *KRAS*-mutant activity in a large number of patients with metastatic NCLSC by interfering with downstream pathways.

Since *KRAS* was found to be involved in lipogenesis inducing fatty acid synthesis [32], different phase I trials demonstrated promising preliminary results of fatty acid synthase (FASN) inhibitors in *KRAS*-mutant NSCLC [33,34,35]. Falchook et al. studied TVB-2640 in a phase I trial, the first-in-class FASNs, aiming to investigate the safety of this small molecule as a monotherapy and in combination with paclitaxel or docetaxel [33]. When TVB-2640 was administered as a monotherapy, the DCR was 42%, and no patient had a complete (CR) or partial response (PR). In combination with paclitaxel, the PR rate was 11%, and the DCR was 70%. A phase II trial is ongoing to determine the response rate of TVB-2640 in *KRAS*-mutant NSCLC patients by examining the RECIST and toxicity profile (ClinicalTrials.gov Identifier: NCT03808558).

Selumetinib, a MEK pathway inhibitor, was tested in the phase II Selected-1 trial. The study included more than 500 patients previously treated, and the association of MEK inhibition and chemotherapy did not improve PFS compared with docetaxel alone in *KRAS*-mutated NSCLC (3.9 vs. 2.8 months; HR 0.93, *p* = 0.44); the median OS was 8.7 months with selumetinib + docetaxel and 7.9 months with placebo + docetaxel (HR, 1.05; *p* = 0.64) [36].

A preclinical trial revealed different activity of the MEK inhibitor selumetinib using murine models with co-occurring *KRAS* mutations. The study proved that the concomitant loss of either *TP53* or *Lkb1* (also known as *STK11*) reduced the response of *KRAS*-mutant cancers to docetaxel monotherapy. The addition of selumetinib improved results in mice with *KRAS*-mutant cancers and *TP53* co-mutations, while those with *KRAS* and *STK11* co-mutations developed primary resistance to this combination therapy [37].

Similarly, trametinib, another MEK inhibitor, showed a similar PFS and response rate in patients with previously treated *KRAS*-mutant NSCLC, randomized to receive the MEK inhibitor or chemotherapy with docetaxel in a phase II trial [38].

The MAPK paradox explained that anti-BRAF agents in *KRAS*-mutated cancers are contraindicated because of the activation of tumorigenesis by the binding to BRAF [39]. Heidorn et al. demonstrated that the inhibition of BRAF in the presence of oncogenic or growth factor-activated RAS induces BRAF binding to CRAF (a type of RAF protein), leading to CRAF hyperactivation of this signaling pathway.

Therefore, other targets were explored. Sorafenib is an oral RAF inhibitor tested in the MISSION, a phase III, placebo-controlled trial conducted in molecularly unselected relapsed or refractory non-squamous NSCLC who failed at least two previous lines [40]. PFS was significantly longer in the *KRAS*-mutant subpopulation than in the *KRAS* wild-type group (2.6 versus 1.7 months; HR, 0.46; *p* = 0.007). However, the PFS advantage did not translate into a survival gain. Indeed, median OS was similar in the sorafenib and placebo groups with *KRAS* mutations (6.4 versus 5.1 months; HR, 0.76; *p* = 0.279) and wild-type *KRAS* (11.0 versus 9.1 months; HR, 0.79; *p* = 0.078).

Research on the PI3K/AKT/mTOR pathway showed that inhibiting those alterations with monotherapy inhibitors might not be sufficient. In a phase II trial, 79 patients received ridaforolimus, an oral inhibitor of mTOR [41]. The ORR, expressed by the complete response and partial response, at 8 weeks was 1%, and no significant benefit in OS was shown. Post-translational modification of *KRAS* promotes the membrane localization of this protein and permits *KRAS* signaling.

**Table 2:** Indirect KRAS inhibition—targeting *KRAS-*regulated pathways.

Target	Therapeutic Drug	Trial	Patients (n)	Overall Response Rate	Ref.
Fatty acid synthase	TVB-2640	Phase I	31	0	[33]
RAF/MEK/ERK pathway inhibition	Selumetinib + docetaxel	Phase III	510	20%	[36]
	Trametinib	Phase II	129	12%	[38]
	Sorafenib	Phase III	703	2.9%	[40]
PI3K/AKT/mTOR pathway inhibition	Buparlisib	Phase II	63	3%	[42]
	Ridaforolimus	Phase II	79	1%	[41]

## 6. Immunotherapy in *KRAS*-Mutant NSCLC

The treatment landscape of metastatic NSCLC has been changed with the advent of monoclonal antibodies targeting PD-1 and its main ligand, PD-L1 [43,44,45,46]. Based on a subgroup analysis, in the CheckMate 057 trial, patients with *KRAS*-mutant lung cancers achieved the greatest OS benefit when comparing immune checkpoint inhibitors (ICIs) with chemotherapy (HR 0.52, 95% CI: 0.29–0.95) [43]. In the OAK study, a phase III clinical trial, the subgroup of patients with *KRAS* mutation also benefitted from immunotherapy with atezolizumab in terms of OS (HR = 0.71; 95% CI: 0.38–1.35) [45].

*KRAS*-mutant NSCLC is the target of interest for PD-1/PD-L1 inhibition for the following reasons: *KRAS*-mutant lung cancers are typically smoking-associated tumors, and are therefore often associated with a high mutational burden [47,48]; these cancers frequently show abundant T-cell infiltration; PD-L1 expression is present in approximately 24–55% of *KRAS*-mutant lung adenocarcinomas [49,50].

Nevertheless, *KRAS*-mutant lung cancers respond differently to immunotherapy and show differences in terms of the immunogenic profile [18].

As previously underlined, *KRAS* co-occurring mutations are fundamental factors determining distinct immune phenotypes within *KRAS*-mutant cancers. *KRAS*/*STK11* co-mutations represent 25% of *KRAS*-mutant NSCLC, characterized by the low presence of tumor-infiltrating lymphocytes and reduced immune markers and PD-L1 levels. *STK11* mutations constitute a major genomic driver of primary resistance to immune checkpoint inhibitors [19]. In a subgroup of patients with *KRAS*-mutant NSCLC in the phase III CheckMate 057 trial, patients with *KRAS*/*STK11* co-mutations demonstrated significantly lower response rates than patients with *KRAS*/*TP53* adenocarcinomas [19]. *KRAS*-mutant lung adenocarcinomas associated with *KEAP1* mutational inactivation demonstrated lower expression rates of PD-L1 and other immune markers, proving refractory to anti–PD-1 antibody therapy [51,52]. Co-mutation in *KEAP1* was also associated with shorter OS from the start of immune therapy in *KRAS*-mutant metastatic NSCLC [53]. *KRAS/p53* co-mutations are associated with significant treatment responses and major susceptibility to immunotherapies, showing improvement in progression-free and overall survival [19]. These observations are based on the *KRAS/p53* immune profile, which is characterized by an inflammatory response [21,53].

Maugeri-Saccà and colleagues explored intratumor heterogeneity and immunologic features among *KEAP1/TP53*-based subtypes in both blood-based NGS and tissue-based NGS cohorts. *KEAP1* single mutant tumors had the shortest survival; the subgroup represented by *KEAP1/TP53* double mutant had an intermediate prognosis similarly to the pure *TP53*-mutant subgroup; and the double wild-type group showed the longest survival [54].

A retrospective exploratory analysis of the IMpower150 phase III study showed that survival benefits, regardless of the treatment combination, were greater in the *KRAS* mutant and *KEAP1/STK11* wild-type population compared to *mKRAS* and co-mutations in *STK11* and/or *KEAP1*, suggesting both a prognostic and predictive value. Moreover, tumors presenting co-mutations in *KRAS* and *TP53* had elevated PD-L1 expression. In contrast, *mKRAS* and co-occurring *STK11-* and *KEAP1*-mutant tumors had reduced PD-L1 expression, suggesting that the addition of bevacizumab to atezolizumab may represent the preferred option for *KRAS* and *TP53* co-mutated NSCLC [55].

An interesting treatment direction is to explore combination therapy using *KRAS* G12C inhibitors and immunotherapy. In a preclinical study, a durable response was produced in immune-competent mice with the administration of AMG510, resulting in the infiltration of tumors by CD8+ T cells and the development of a pro-inflammatory tumor microenvironment [56]. Several studies are underway to evaluate the synergistic activity between *KRAS* G12C inhibitors and anti PD-1/PD-L1 therapy (ClinicalTrials.gov Identifier: NCT03785249, NCT04185883, NCT03600883, NCT04613596).

## 7. CDK4/6 and SHP2 Inhibition

The description of a biological connection between *KRAS* and cyclin-dependent kinase (CDK)4/6 was observed in preclinical studies, suggesting that the robust and selective inhibition of CDK4/6 could respond to NSCLC patients carrying *KRAS* oncogenes [57,58].

Nevertheless, the JUNIPER trial [59] and the SWOG S1400C study [60], which tested the efficacy of abemaciclib and palbociclib as single agents, did not show great activity of CDK 4/6 inhibitors in mutant *KRAS* NSCLC tumors (Table 3).

## 8. Direct KRAS G12C Inhibition

The complex challenge of directly targeting the KRAS protein is highly connected to the unicity of this protein, characterized by a shallow surface, high affinity for GTP molecules and lack of active binding sites [63,64,65].

Developments in the field of crystallography facilitated the study of new molecules able to interact with the KRAS protein and its specific conformation [66]; with the aim of finding potent Switch II pocket inhibitors, several molecules were tested in vitro and in vivo (Table 4).

Compound 12 had suboptimal pharmacologic properties, proved by the fact that the compound did not engage covalently *KRAS* G12C mutant cells [66,67]. Other molecules proved more potent and efficient in inhibiting *KRAS* mutant cells in vitro. ARS-1620 exhibited in vitro and in vivo potency and high selectivity for *KRAS* G12C [68].

Ostrem et al. demonstrated that small compounds could covalently bind to the switch II pocket of *KRAS* G12C; therefore, the numerous efforts with the aim of discovering this new pocket facilitated the direct targeting of *KRAS*. Recently, two molecules directly targeting *KRAS* G12C reached phase II/III trials. Their mechanism of action is based on the conversion of the preference of *KRAS* from GTP to GDP, holding a KRAS state from an active to inactive GDP-bound form and interrupting intracellular signaling and tumor cell growth [66].

Sotorasib (AMG 510) is an irreversible *KRAS* G12C inhibitor, which provided encouraging results in terms of response rate (RR) and duration of response (DOR) [69]. CodeBreaK 100 is a multicentre, single-arm, open-label clinical trial (ClinicalTrials.gov Identifier: NCT03600883) evaluating sotorasib alone and in combination with anti-PD-1/PD-L1, including patients with locally advanced or metastatic NSCLC with *KRAS* G12C mutations. Patients showed progression of the disease after three or fewer lines of therapy, including ICIs targeting PD-1/PD-L1, platinum-based combination chemotherapy or targeted therapy if they harbored *EGFR*, *ALK*, or *ROS1* alterations.

In the phase I monotherapy arm of CodeBreaK 100, 59 patients with NSCLC achieved an ORR of 32.2%, median duration of response of 10.9 months and a median progression-free survival of 6.3 months [69,70,71,72]. The dose escalation of 960 mg twice daily proved to be active and safe. The drug was well tolerated, with a grade 3/4 TRAE rate of 11.6%.

The phase II trial evaluated the efficacy and safety of sotorasib in a patient with locally advanced or metastatic *KRAS* G12C NSCLC who was previously treated with standard therapies. Among the 126 enrolled patients, Skoulidis et al. demonstrated an RR of 37.2%, a median PFS of 6.3 months and a median DOR of 10 months [73].

In May 2021, the US FDA granted accelerated approval to sotorasib based on CodeBreaK 100 for patients with NSCLC who received at least one prior line of anti-cancer therapy (immunotherapy and/or chemotherapy). This small molecule became the first drug targeting *KRAS* to be approved for treating locally advanced or metastatic NSCLC with *KRAS* G12C mutation [74].

The CodeBreak 101 study (ClinicalTrials.gov Identifier: NCT04185883) investigated sotorasib monotherapy and in combination with other anti-cancer treatments in patients with advanced solid tumors with *KRAS* G12C mutation. The CodeBreaK 200 study is a phase III trial comparing docetaxel to sotorasib in patients with *KRAS* G12C mutation in the second-line setting (ClinicalTrials.gov Identifier: NCT04303780). Skoulidis and colleagues conducted an exploratory analysis of the phase II trial that evaluated the activity of sotorasib and investigated the association between mutations in *STK11*, *KEAP1* and *TP53* and their response to sotorasib treatment. In the subgroup with mutated *STK11* and wild-type *KEAP1*, a benefit in terms of response was seen in 50% of the patients. Patients with both mutated *STK11* and *KEAP1* showed a response of 23%; otherwise, wild-type *STK11* and mutated *KEAP1* demonstrated a response of 14%. Future prospective studies could validate the identification of subgroups of patients who may benefit differently from sotorasib treatment [75].

Adagrasib (MRTX 849), another small molecule, was studied in the phase I-II KRYSTAL-1 clinical trial (ClinicalTrials.gov Identifier: NCT03785249) recruiting patients with pre-treated advanced or metastatic solid tumors [76,77].

Recently, Jänne et al. published the results from cohort A of the phase I/II KRYSTAL-1 trial with single agent adagrasib in previously treated patients with *KRAS* G12C-mutated NSCLC [78]. After a median follow-up of 12.9 months, the objective response was 43%, with a disease control rate of 80% among the 112 patients with measurable disease at baseline. The median duration of response was 8.5 months, with 50% of NSCLC patients remaining on treatment. The median PFS was 6.5 months, and the median OS was 12.6 months, with 6- and 12-month OS rates of 71% and 51%, respectively. The authors evaluated the efficacy among 33 patients with treated, stable central nervous system (CNS) metastases. The intracranial ORR was 33%, with a DCR of 85%; the median intracranial PFS was 5.4 months. The treatment was well-tolerated, with 43% of patients experiencing grade 3 or 4 adverse events.

Subsequent clinical trials are currently investigating adagrasib as monotherapy or in association with other compounds in patients with advanced or metastatic solid tumors. The phase III KRYSTAL-12 study is recruiting pre-treated patients with NSCLC to MRTX 849 versus docetaxel [79]. In the KRYSTAL-1 trial, a subgroup analysis was performed with the aim of exploring co-mutations in *STK11*, *KEAP1*, *P53* and *CDKN2A*. There were no differences in these subgroups except for patients with *STK11* wild-type, *KEAP1*-mutant disease, which presented inferior response rates (only one of seven patients had a response) [80]. The phase II trial Lung-MAP S1900E with sotorasib (NCT04625647) will further clarify the impact of co-mutations on the activity of KRAS G12C inhibitors.

## 9. Resistance to KRAS G12C Inhibitor

**Figure 2:**
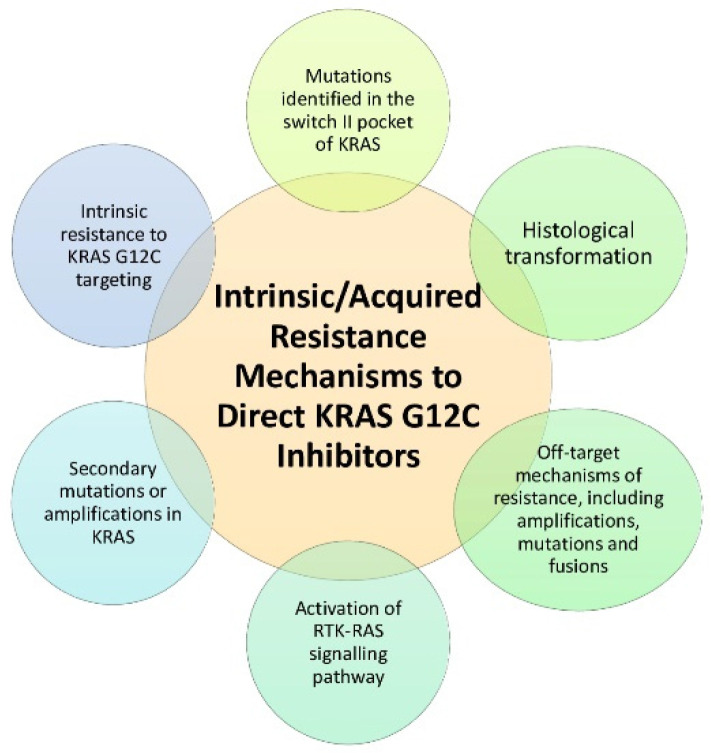
Mechanisms of resistance to direct KRAS G12C inhibitors.

Despite the evident clinical benefit, underlying mechanisms lead to the development of resistance in most patients treated with KRAS G12C inhibitors (Figure 2).

As evidenced by clinical studies, KRAS G12C inhibitors did not determine significant tumor shrinkage in about half of the patients included in clinical trials. Furthermore, disease progression was evidenced in about 10% of patients. During the treatment with target therapy, the emergence of resistance mechanisms in cancer cells can determine the progression of the disease after an initial response or stability of the disease.

Intercellular variability and intratumoral heterogeneity are recognized as the most prominent causes of resistance to KRAS G12C inhibitors; nevertheless, multiple resistance mechanisms are described, leading to the idea that combination strategies and a more personalized therapy are necessary to overcome resistances.

Resistance to KRAS G12C inhibitors can be either intrinsic or acquired. It has been suggested that putative resistance mechanisms could lead to intrinsic resistance to KRAS G12C targeting; the presence of non-*KRAS* G12C mutations may be evident at baseline, leading to significant clinical implications for patients eligible for KRAS G12 inhibitors [81].

Two main types of biological mechanisms of adaptative resistance are described, namely on-target resistance, determined by mutations or amplification in KRAS cells, and off-target resistance, with the activation of another oncogenic signaling pathway.

Ryan et al. examined the adaptive feedback response to KRAS G12C inhibition and observed evidence of rapid RAS pathway reactivation in most KRAS G12C models. These data showed that vertical pathway inhibition strategies, such as combinations of KRAS G12C inhibitors with SHP2 inhibitors, may be effective in interrupting feedback reactivation of the RAS pathway following KRAS G12C inhibition and may represent a possible therapeutic approach for KRAS G12C cancers [82].

As already mentioned, a phase I/II clinical trial (NCT04330664) tested the combination of adagrasib and TNO-155 (investigational SHP-2 inhibitor) and several other trials of SHP2 inhibitors alone or in combination with other drugs are currently ongoing.

The nuclear factor NRF2 (erythroid 2-like 2, NFE2L2), regulated by the KEAP1 protein, is a key transcription factor in the cellular antioxidant response. In addition to predicting poor response to checkpoint inhibitor immunotherapy [53], KEAP1 or NRF2 mutations may also be related to resistance to KRAS inhibitors, such as adragasib [83].

A recent clinical study evaluated 38 patients, of which 27 with NSCLC with *KRAS* G12C-mutant cancers were treated with adagrasib monotherapy [84]. The authors analyzed genetic variations in circulating tumor DNA or tissue samples from patients resistant to adagrasib through next-generation sequencing. In total, 42% of those patients showed a putative resistance mechanism to adagrasib, including secondary mutations or amplifications to *KRAS*; the activation of the RTK–RAS signaling pathway; and the histological transformation of adenocarcinoma to squamous cell carcinoma. Mutations identified in the switch II pocket of *KRAS* may impede the binding of KRAS inhibitors. In addition, Awad et al. identified acquired resistance mechanisms, including *MET* amplification; activating mutations in *NRAS*, *BRAF*, *MAP2K1*, and *RET*; oncogenic fusions involving *ALK*, *RET*, *BRAF*, *RAF1*, and *FGFR3*; and mutations with loss-of-function in *NF1* and *PTEN.*

Recently, Tanaka et al. described acquired resistance to adagrasib in an NSCLC patient with 10 secondary genetic mutations on the RAS–RAF–MEK–ERK pathway [85]. Among them, a new *KRAS* Y96D mutation was observed directly affecting the switch-II pocket, resulting in resistance to sotorasib, adagrasib or ARS-1620 across multiple models. Thereby, the authors suggested that the previously undescribed Y96D mutation may have a novel and specific role in driving resistance to KRAS G12C inhibitors [85]. RM-018 is a novel KRAS G12C inhibitor that binds specifically to the GTP-bound, active state of KRAS G12C. Analyzing the different mechanisms of action of this class of inhibitor, they hypothesized that RM-018 could have the ability to bind and inhibit KRAS G12C/Y96D and may represent a potential therapeutic strategy to overcome this acquired resistance mechanism [85].

Patients treated with KRAS G12C-targeted therapies can be affected by intrinsic and acquired resistance co-occurring in the same patient [86]. Combining KRAS G12C inhibitors with other treatments could improve the clinical response and overcome resistances.

Several mechanisms can contribute to the development of resistance to KRAS G12C inhibitors. The results of various studies highlight the need to develop KRAS inhibitors capable of overcoming resistances, determine the appropriate sequence of treatments and develop effective combination therapy regimens.

## 10. Open Questions and Future Challenges

The efficacy of KRAS G12C inhibitors could be enhanced by the combination of treatments with targeted agents, immune checkpoint inhibitors, and downstream KRAS inhibitors; several studies are ongoing to evaluate the clinical efficacy and safety of combination therapy.

In recent years, numerous efforts have been made to implement therapeutic possibilities for patients with *KRAS*-mutant NSCLC. Several specific drugs have been introduced into preclinical and current clinical practice.

The position of these inhibitors in the *KRAS* G12C lung cancer treatment strategy algorithm is still evolving; primarily, the activity of *KRAS* G12C may be limited due to the genetic heterogeneity and complex biology of *KRAS*.

A multiplex assay for mutations and fusion genes with next-generation sequencing (NGS) is essential for the appropriate selection of patients, as recommended by ESMO Clinical Practice Guidelines [3]. A possible approach is to treat patients unsuitable for standard first-line therapy with direct KRAS G12C inhibitors. Co-mutations of *KRAS* G12C and *STK11* provided a poorer response to ICI therapy. Therefore, treatment with KRAS G12C inhibitors could represent a valid option in this first-line setting. Another strategy could be the association of KRAS G12C inhibitors with ICI and/or chemotherapy.

Overcoming the mechanisms of innate/acquired resistance that reduce the clinical efficacy of *KRAS* G12C inhibition constitutes a future challenge to obtaining a durable response. Combining KRAS G12C inhibitors with other therapy strategies, such as immunotherapy or KRAS pathway inhibitors, as previously described, could improve clinical benefits and overcome resistances.

Co-occurring genetic alterations and the mutant *KRAS* allele copy number gains define a variety of tumor microenvironments, which lead similar histological tumors of different drug sensitivities. Individualized treatments are needed to overcome the biological heterogeneity of *KRAS*-mutant NSCLC and ensure the development of new effective treatment strategies.

In addition, further developments are warranted to identify selective agents against *KRAS* G12D and G12V mutations, representing 38% of all *KRAS*-mutant lung adenocarcinomas. It is necessary to develop selective inhibitors against single mutations or pan-KRAS inhibitors able to block all mutant proteins.

Furthermore, many questions remain, and additional studies are needed to optimize these therapeutic strategies in the clinical setting; however, recent results are encouraging and demonstrate the potential to adequately treat patients with *KRAS*-mutant NSCLC. A major concern is identifying patients who can potentially benefit from *KRAS*-targeted monotherapies or combination therapy strategies.

## Figures and Tables

**Table 3:** Indirect KRAS inhibition—Immune checkpoint, CDK4/6 and SHP2 inhibition.

Target	Therapeutic Drug	Trial	Patients (n)	Overall Response Rate	Ref.
Immune checkpoint inhibition	Nivolumab	EAP	324	20%	[60]
CDK inhibition	Abemaciclib vs. erlotinib	Phase III	270	9%	[59]
	Palbociclib	Phase II	53	6%	[61]
SHP2 inhibition	RMC 4630	Phase I	18	11%	[62]

**Table 4:** Therapeutic strategies—Direct KRAS G12C inhibition.

Target	Therapeutic Drug	Trial	Patients (n)	Overall Response Rate	Ref.
Protein-based inhibition (KRAS binders)	AMG 510/sotorasib	Phase I–III	129	32.2%	NCT03600883 CodeBreak 100
	MRTX 849/adragasib	Phase I–III	79	45%	NCT03785249 KRYSTAL-1 trial
	JNJ-74699157	Phase I	140		NCT04006301
	GDC-6036	Phase I			NCT04449874
	D-1553	Phase I			NCT04585035

## Data Availability

Not applicable.
